# The impact of regional socioeconomic deprivation on the timing of HIV diagnosis: a cross-sectional study in Germany

**DOI:** 10.1186/s12879-022-07168-x

**Published:** 2022-03-17

**Authors:** Annemarie Pantke, Jens Hoebel, Matthias an der Heiden, Niels Michalski, Barbara Gunsenheimer-Bartmeyer, Kirsten Hanke, Norbert Bannert, Viviane Bremer, Uwe Koppe

**Affiliations:** 1 Department of Infectious Disease Epidemiology, Robert Koch Institute, Berlin, Germany; 2 Institute of Public Health, Charité – Universitätsmedizin Berlin, Berlin, Germany; 3 Department of Epidemiology and Health Monitoring, Robert Koch Institute, Berlin, Germany; 4 Department of Infectious Diseases, Robert Koch Institute, Berlin, Germany

**Keywords:** HIV recency testing, AIDS, Socioeconomic factors, Social inequalities, Heterosexuals, Men who have sex with men

## Abstract

**Background:**

HIV infections which are diagnosed at advanced stages are associated with significantly poorer health outcomes. In Germany, the proportion of persons living with HIV who are diagnosed at later stages has remained continuously high. This study examined the impact of regional socioeconomic deprivation on the timing of HIV diagnosis.

**Methods:**

We used data from the national statutory notification of newly diagnosed HIV infections between 2011 and 2018 with further information on the timing of diagnosis determined by the BED-Capture-ELISA test (BED-CEIA) and diagnosing physicians. Data on regional socioeconomic deprivation were derived from the German Index of Socioeconomic Deprivation (GISD). Outcome measures were a non-recent infection based on the BED-CEIA result or an infection at the stage of AIDS. The effect of socioeconomic deprivation on the timing of diagnosis was analysed using multivariable Poisson regression models with cluster-robust error variance.

**Results:**

Overall, 67.5% (n = 10,810) of the persons were diagnosed with a non-recent infection and 15.2% (n = 2746) with AIDS. The proportions were higher among persons with heterosexual contact compared to men who have sex with men (MSM) (76.8% non-recent and 14.9% AIDS vs. 61.7% non-recent and 11.4% AIDS). MSM living in highly deprived regions in the countryside (< 100 k residents) were more likely to have a non-recent infection (aPR: 1.16, 95% CI: 1.05–1.28) as well as AIDS (aPR: 1.41, 95% CI: 1.08–1.85) at the time of diagnosis compared to MSM in less deprived regions in the countryside. No differences were observed among MSM from towns (100 k ≤ 1 million residents) or major cities (≥ 1 million residents), and no differences overall in the heterosexual transmission group.

**Conclusions:**

An effect of socioeconomic deprivation on the timing of HIV diagnosis was found only in MSM from countryside regions. We suggest that efforts in promoting HIV awareness and regular HIV testing are increased for heterosexual persons irrespective of socioeconomic background, and for MSM with a focus on those living in deprived regions in the countryside.

**Supplementary Information:**

The online version contains supplementary material available at 10.1186/s12879-022-07168-x.

## Background

The timing of an HIV diagnosis can vary widely across individuals. An undiagnosed and/or untreated infection advances with a progressive depletion of CD4 cells and a continuous decrease in immune functioning [1]. This progression can be prevented by antiretroviral therapy (ART) in most patients [2]. Late HIV presentation leading to a deferred commencement of ART therefore poses severe health risks to the individual and is also concerning on the population health level.

Persons with a delayed start of ART have a higher risk of developing AIDS-defining illnesses and experience increased morbidity compared to people with an early start of ART [3, 4]. Late presentation is also associated with substantially higher medical health care costs resulting from increased treatment needs of persons with advanced HIV infections [5, 6]. On the population health level, delayed diagnoses are concerning as they pave the way for further transmission through individuals who are unaware of their status [7]. An early diagnosis together with continuous treatment furthermore plays a pivotal role in controlling HIV transmission in populations as effective ART leads to viral suppression and the practical elimination of a risk for onward sexual transmission [8].

Despite continuous efforts with respect to awareness campaigns and an increased focus on testing [9], in Europe an estimated 40–60% of persons are diagnosed with a CD4 cell count below 350 cells/mm^3^ and/or have experienced an AIDS-defining illness at the time of diagnosis [10]. The highest proportions are found in the age groups of 50 years and older and among people who acquired HIV via heterosexual contact [10, 11]. A similar distribution can be observed in Germany [12]. In 2019, around half of the HIV infections were diagnosed at later stages with 34% at the stage of advanced immunodeficiency and 15% with AIDS. These numbers have remained continuously high since the early 2000s [12]. Here, the highest proportions of delayed diagnoses were also found among the older age groups, persons with heterosexual contact as well as migrants from Sub-Saharan Africa and Asia and the Pacific [12, 13].

In order to reduce the burden of late HIV presentation, it is necessary to gain a better understanding of the underlying factors that play a role in HIV health behaviours. Socioeconomic status (SES) is a factor whose impact on health has been widely investigated. Whether measured by poverty, education, social class or other indicators, low SES predicts worse outcomes for a wide range of health behaviours and events [14]. For HIV and AIDS, some studies underline this link, showing that individuals of lower SES are at a disproportionately higher risk for contracting HIV, presenting at later stages and also have higher mortality rates [15–17]. In Germany, this relationship has not been examined yet. As the HIV surveillance system does not include information on socioeconomic factors at the individual patient level, this study utilised the German Index of Socioeconomic Deprivation (GISD), which describes socioeconomic inequalities at different regional levels in Germany [18]. The GISD was generated at the levels of associations of municipalities, administrative districts and administrative regions and is based on the sub-dimensions of education, occupation, and income. The calculated deprivation scores depict the socio-spatial burden in the respective area and can be utilised to investigate the link between regional socioeconomic deprivation and health.

Using the GISD, the objective of this study was to examine whether socioeconomic deprivation in Germany affects the timing of HIV diagnosis. Seeing that HIV support structures are generally better-developed and more frequent in urban areas compared to rural areas [19, 20], we also aimed to investigate the possible role of city size with regard to this relationship. Knowledge about the potential impact of regional socioeconomic deprivation can be used to improve and render public health strategies aimed at promoting HIV awareness and early testing behaviours more effectively.

## Methods

### Study design

This study is a cross-sectional study using data from the “InzSurv-HIV” study at the Robert Koch Institute (RKI) in Germany. The dataset contains pseudonymised information from the national statutory notification of newly diagnosed HIV infections that were directly reported to the RKI. In addition to that, the RKI received dried serum or plasma spots from participating laboratories which allowed to test about two thirds of all new HIV diagnoses for recency of infection using the BED-Capture-ELISA (BED-CEIA) [21, 22]. Infections were classified as recent or non-recent with a cut-off point that corresponds to a mean duration of approximately five months after the infection [23]. The BED-CEIA can produce false-recent results with an estimated false-recent rate (FRR) of 4.3% for HIV-1 subtype B and 28.9% for non-B subtypes [24]. We considered infections classified as recent while diagnosed with an AIDS-defining condition as non-recent, which applied to 2% of the cases. Included in the analysis were all newly diagnosed infections between 2011 and 2018. Excluded were diagnoses of persons with the age below 15 years as well as diagnoses of persons with a transmission mode of blood transfusion, haemophilia, occupational exposition and mother to child transmission as these present rare and uncommon risk groups. The GISD data were linked to this dataset on the basis of the 3-digit ZIP code with each person receiving the respective socioeconomic deprivation score of their residential area. In addition to that, data on city population size were derived from the Federal Statistical Office of Germany and merged to the dataset [25].

### Outcomes and covariates

The risk for late HIV presentation was examined at two time points: The first outcome was a non-recent HIV infection at the time of diagnosis as determined by the BED-CEIA. A non-recent HIV infection was defined as an infection that has been diagnosed after a mean duration of more than approximately 20 weeks (= 5 months) since it was acquired [23]. The second outcome was an HIV infection that was diagnosed at the clinical stage of AIDS. This outcome was derived from the CDC classification system for HIV diagnoses in which category C classifies patients who have already developed an AIDS-defining illness at the time of their HIV diagnosis [26]. The classification was done by the diagnosing physician and reported to the RKI via the statutory notification.

While the first outcome identifies all cases diagnosed after the acute phase, the second outcome is more specific and only looks at diagnoses received at a further advanced stage with an AIDS-defining condition. The two outcome measures depict different points in time with regard to HIV progression and were analysed separately.

The exposure of interest was regional socioeconomic deprivation using the GISD, which is based on the sub-dimensions of education, occupation, and income [18, 27]. The calculated GISD scores which were generated on the basis of regional indicator values for each sub-dimension were first divided into quintiles and then grouped into three categories, representing low deprivation (lowest quintile), medium deprivation (middle three quintiles) and high deprivation (highest quintile). High regional socioeconomic deprivation can be equally understood as a lack of socioeconomic resources or a low socioeconomic status on average among persons living in that particular region [18].

Further variables included in the analysis were transmission mode (men who have sex with men (MSM), persons with heterosexual contact (HET), persons who inject drugs (PWID)), sex (male, female), approximated age at the time of infection (15–19, 20–29, 30–39, 40–49, 50–59, 60–69, > 69 years), region of origin (Western and Central Europe, Asia and the Pacific, Caribbean, Eastern Europe and Central Asia, Latin America, Middle East and North Africa, North America, Sub-Saharan Africa) and city size (countryside: < 100 k residents, town: 100 k—< 1 million residents, major city: ≥ 1 million residents). As the age at the time of infection is unknown, this variable was approximated from the available age at the time of diagnosis by subtracting the approximate median progression periods on the basis of the CDC classification system [26]. We subtracted one age year from persons classified as category A (acute, asymptomatic infection), five age years from persons classified as category B (chronic, symptomatic infection), and ten age years from persons classified as category C (AIDS) [26, 28, 29]. No age years were subtracted from persons younger than 30 years at the time of diagnosis to avoid computing unrealistic outliers in the lower end age groups. Even though the median time periods until the development of AIDS decrease with older age [28], this procedure is justified as persons who are diagnosed with an AIDS-defining illness at a younger age usually have shorter lags between HIV contraction and diagnosis, while persons diagnosed with AIDS in the older age groups tend to have longer lags.

### Statistical analysis

Separate complete case analyses were conducted for both outcome measures. To estimate the effect of socioeconomic deprivation on late presentation, prevalence ratios using multivariable Poisson regressions with ZIP code cluster-robust error variance to account for the individual and regional level data structure were computed. For outcomes that are not rare (prevalence > 10%), Poisson regression models provide conservative and consistent effect estimates and were therefore used in preference to logistic regression [30]. We adjusted the models for the variables of transmission mode, sex, approximated age at the time of infection, region of origin and city size. These variables were identified as confounders by a directed acyclic graph, which was created on the basis of prior research (see  Additional file 1: Figure S1). We performed stratified analyses for MSM and persons with heterosexual contact as effect modification was expected for these groups due to their differing health seeking behaviours with respect to HIV [13]. In the case of discernible effects, an interaction term between the GISD and city size was planned to be included in the models to examine a potential interaction of these variables, seeing that HIV support structures in urban areas differ from those in rural areas [19, 20]. Adjusted prevalence ratios (aPR) and 95% confidence intervals (95% CI) were calculated. All analyses were performed using STATA 15.0 (Stata Statistical Software: Release 15, United States).

### Sensitivity analyses

We conducted sensitivity analyses addressing the missing values of the outcome measures. Baseline characteristics of persons with available vs. missing BED-CEIA results and CDC classification were compared to examine whether persons included in the analyses differed from those who were excluded. Seeing that the categories across the covariates in our analyses overlap, we also conducted sensitivity analyses assessing the distribution of region of origin among persons with heterosexual contact. This was done to account for the fact that migrants from regions with differing HIV prevalences and dynamics make up a substantial proportion of the heterosexual persons who are diagnosed with HIV in Germany [12]. Post-hoc multivariable analyses restricting the population to people of Western and Central European origin were run to investigate if these yield similar results.

### Ethical approval

The “InzSurv-HIV” study has been approved by the data protection officer of the Robert Koch Institute and the Federal Commissioner for Data Protection and Freedom of Information (II-401/008#0016). Ethical approval for the monitoring of recent HIV infections was given by the ethics commission at the Charité – Universitätsmedizin Berlin (EA1/007/08). No patient informed consent was obtained as the data on new HIV diagnoses were derived from the national statutory HIV surveillance which is carried out in compliance with the German Infection Protection Act (IfSG). The samples used in the BED-CEIA were residuals from routine diagnostic processing. Recency testing of these samples is only licensed for epidemiological analyses and does not allow for individual patient analyses. All methods used in our study were carried out in accordance with relevant guidelines and regulations.

## Results

A total of 16,010 persons with BED-CEIA results and 18,092 persons with a documented CDC classification were included in the analysis (see Additional file 2: Figure S2). Our sensitivity analyses addressing the missing data of the outcome measures showed that included and excluded persons were generally comparable with regard to baseline characteristics (see Additional file 3: Tables S1 and S2). Among the 16,010 persons with available BED-CEIA results, 67.5% had an HIV infection classified as non-recent at the time of diagnosis (Table 1). Persons in the highest socioeconomic deprivation quintile showed a slightly higher proportion of non-recent infections (69.8%) compared to the low (68.0%) and medium deprivation quintiles (67.2%). Concerning the other variables included in the analysis, the highest proportions of non-recent infections were found in the heterosexual transmission group (76.8%), persons between 60 and 69 years at the time of diagnosis (76.2%), migrants from Sub-Saharan Africa (79.1%) and among persons living in the countryside (70.3%). Among the 18,092 persons with a documented CDC classification, 15.2% had reached the clinical stage of AIDS at the time of their diagnosis (Table 1). Persons living in regions with the highest socioeconomic deprivation were more often diagnosed with an AIDS-defining condition (19.4%) than persons of low (16.1%) and medium deprivation (15.3%) background. With regard to the other variables, the highest proportions of AIDS-defining conditions were observed in the heterosexual transmission group (14.9%), persons older than 69 years at the time of diagnosis (37.2%), migrants from Asia and the Pacific (21.6%) as well as persons living in the countryside (17.7%) (Table 1).

**Table 1:** Baseline data of newly diagnosed HIV infections by BED-CEIA^1^ result and CDC classification

	N = 16,010		N = 18,092	
	Non-recent infection	Recent infection	AIDS^2^	No AIDS^2^
Total	10,810 (67.5%)	5200 (32.5%)	2746 (15.2%)	15,346 (84.8%)
GISD^3^				
Low deprivation	2119 (68.0%)	997 (32.0%)	635 (16.1%)	3305 (83.9%)
Medium deprivation	4969 (67.2%)	2427 (32.8%)	1375 (15.3%)	7594 (84.7%)
High deprivation	844 (69.8%)	366 (30.2%)	321 (19.4%)	1333 (80.6%)
Missing	2878 (67.1%)	1410 (32.9%)	415 (11.8%)	3114 (88.2%)
Transmission mode				
MSM^4^	5482 (61.7%)	3397 (38.3%)	1161 (11.4%)	9001 (88.6%)
HET^5^	2951 (76.8%)	893 (23.2%)	633 (14.9%)	3619 (85.1%)
PWID^6^	350 (63.9%)	198 (36.1%)	66 (9.7%)	612 (90.3%)
Missing	2072 (74.0%)	712 (26.0%)	886 (29.5%)	2114 (70.5%)
Sex				
Male	8486 (65.7%)	4437 (34.3%)	2233 (15.2%)	12,496 (84.8%)
Female	2305 (75.2%)	760 (24.8%)	512 (15.3%)	2841 (84.7%)
Missing	19 (86.4%)	3 (13.6%)	1 (10.0%)	9 (90.0%)
Age (time of diagnosis)				
15 to 19	162 (53.5%)	141 (46.5%)	13 (4.5%)	274 (95.5%)
20 to 29	2676 (62.2%)	1627 (37.8%)	236 (5.3%)	4246 (94.7%)
30 to 39	3413 (67.2%)	1667 (32.8%)	683 (12.1%)	4955 (87.9%)
40 to 49	2595 (71.2%)	1052 (28.8%)	871 (20.0%)	3493 (80.0%)
50 to 59	1377 (73.7%)	492 (26.3%)	607 (26.3%)	1702 (73.7%)
60 to 69	428 (76.2%)	134 (23.8%)	254 (34.1%)	492 (65.9%)
> 69	123 (68.7%)	56 (31.3%)	81 (37.2%)	137 (62.8%)
Missing	36 (53.7%)	31 (46.3%)	1 (2.1%)	47 (97.9%)
Approx. age (time of infection)				
15 to 19	162 (53.5%)	141 (46.5%)	13 (4.5%)	274 (95.5%)
20 to 29	4019 (68.7%)	1834 (31.3%)	919 (15.0%)	5202 (85.0%)
30 to 39	3257 (67.0%)	1608 (33.0%)	871 (15.5%)	4755 (84.5%)
40 to 49	2211 (68.7%)	1009 (31.3%)	607 (15.6%)	3289 (84.4%)
50 to 59	832 (66.4%)	421 (33.6%)	254 (16.2%)	1312 (83.8%)
60 to 69	250 (68.9%)	113 (31.1%)	77 (16.7%)	383 (83.3%)
> 69	43 (50.0%)	43 (50.0%)	4 (4.6%)	84 (95.4%)
Missing	36 (53.7%)	31 (46.3%)	1 (2.1%)	47 (97.9%)
Region of origin				
Western and Central Europe	7070 (64.7%)	3852 (35.3%)	2054 (15.7%)	11,040 (84.3%)
Asia and the Pacific	334 (71.7%)	132 (28.3%)	108 (21.6%)	393 (78.4%)
Caribbean	46 (64.8%)	25 (35.2%)	6 (8.5%)	65 (91.5%)
Eastern Europe and Central Asia	481 (72.0%)	187 (28.0%)	94 (13.0%)	631 (87.0%)
Latin America	218 (64.7%)	119 (35.3%)	31 (8.5%)	334 (91.5%)
Middle East and North Africa	245 (70.4%)	103 (29.6%)	42 (11.3%)	327 (88.6%)
North America	44 (64.7%)	24 (35.3%)	10 (14.9%)	57 (85.1%)
Sub-Saharan Africa	1745 (79.1%)	460 (20.9%)	274 (12.3%)	1949 (87.7%)
Missing	627 (67.8%)	298 (32.2%)	127 (18.8%)	550 (81.2%)
City size				
Countryside < 100 k residents	4439 (70.3%)	1880 (29.7%)	1292 (17.7%)	6003 (82.3%)
Town 100 k—< 1 million residents	3429 (66.4%)	1732 (33.6%)	854 (14.8%)	4871 (85.2%)
Major city > = 1 million residents	2910 (64.9%)	1571 (35.1%)	588 (11.9%)	4417 (88.1%)
Missing	32 (65.3%)	17 (34.7%)	10 (15.4%)	55 (84.6%)

^1^*BED-CEIA* BED-Capture-ELISA recency test, ^2^*AIDS* Evidence of AIDS-defining illness, ^3^*GISD* German Index of Socioeconomic Deprivation, ^4^*MSM* Men who have sex with men, ^5^*HET* Persons with heterosexual contact, ^6^*PWID* Persons who inject drugs

In the stratified multivariable analyses of the BED-CEIA outcome measure, a total of 6511 MSM and 2491 heterosexual persons (1704 women and 787 men) were included (see Additional file 2: Figure S2). Among MSM living in regions that belong to the highest socioeconomic deprivation quintile, we observed a weak increase in the prevalence of non-recent infections at the time of diagnosis (aPR: 1.06, 95% CI: 0.99–1.13) compared to MSM in regions of lower deprivation (Table 2). In the heterosexual transmission group, no differences between persons of high, medium or low socioeconomic deprivation background were observed (Table 2). An interaction term between the GISD and city size was included in the regression model of the MSM stratum, which showed that these variables interact with each other. While MSM from highly and medium deprived countryside areas had a significant increase in the prevalence of non-recent HIV infections at the time of diagnosis (aPR: 1.16, 95% CI: 1.05–1.28; aPR: 1.09, 95% CI: 1.01–1.18) compared to MSM from countryside areas of low socioeconomic deprivation (Table 3), no meaningful differences in the adjusted prevalence ratios were observed between MSM from towns or major cities with different deprivation levels (Table 3).

**Table 2:** Multivariable analysis of non-recent HIV infections stratified by transmission mode

n = 2491				n = 6511		
		MSM^2^			HET^3^	
	n (%)^†^	aPR [95% CI]^‡^	*p*-value	n (%)^†^	aPR [95% CI]^‡^	*p*-value
GISD^1^						
Low deprivation	1052 (61.7%)	1		556 (76.9%)	1	
Medium deprivation	2611 (62.1%)	1.00 [0.96, 1.05]	0.873	1161 (76.5%)	1.00 [0.95, 1.05]	0.940
High deprivation	398 (66.0%)	1.06 [0.99, 1.13]	0.091	188 (74.9%)	0.98 [0.90, 1.05]	0.535
Sex						
Male	4061 (62.4%)	1		618 (78.5%)	1	
Female		(omitted)		1287 (75.5%)	0.95 [0.91, 1.00]	0.030
Approx. age (time of infection)						
15 to 19	43 (38.1%)	0.60 [0.47, 0.76]	< 0.001	49 (76.6%)	1.00 [0.88, 1.15]	0.919
20 to 29	1485 (61.6%)	0.98 [0.93, 1.02]	0.342	703 (79.3%)	1.04 [0.99, 1.09]	0.129
30 to 39	1235 (62.8%)	1		632 (76.1%)	1	
40 to 49	935 (66.1%)	1.05 [1.00, 1.10]	0.044	310 (74.9%)	0.99 [0.93, 1.07]	0.854
50 to 59	275 (59.9%)	0.95 [0.88, 1.03]	0.185	156 (73.2%)	0.99 [0.90, 1.08]	0.769
60 to 69	74 (62.7%)	1.00 [0.85, 1.17]	0.987	43 (68.3%)	0.92 [0.78, 1.09]	0.333
> 69	14 (50.0%)	0.80 [0.58, 1.11]	0.181	12 (63.2%)	0.86 [0.60, 1.24]	0.416
Region of origin						
Western and Central Europe	3685 (62.2%)	1		631 (70.8%)	1	
Asia and the Pacific	103 (65.6%)	1.07 [0.96, 1.20]	0.222	89 (81.7%)	1.16 [1.04, 1.28]	0.007
Caribbean	14 (70.0%)	1.14 [0.84, 1.54]	0.407	11 (68.8%)	0.98 [0.70, 1.37]	0.889
Eastern Europe and Central Asia	44 (57.1%)	0.93 [0.77, 1.12]	0.433	109 (77.3%)	1.08 [0.98, 1.20]	0.107
Latin America	105 (66.0%)	1.09 [0.98, 1.20]	0.101	13 (68.4%)	0.97 [0.71, 1.32]	0.838
Middle East and North Africa	49 (62.8%)	1.02 [0.85, 1.23]	0.802	54 (80.6%)	1.11 [0.98, 1.25]	0.089
North America	24 (72.7%)	1.19 [0.92, 1.54]	0.191	1 (50.0%)	0.69 [0.17, 2.90]	0.616
Sub-Saharan Africa	37 (60.7%)	1.01 [0.82, 1.25]	0.911	997 (80.0%)	1.12 [1.06, 1.18]	< 0.001
City size						
Countryside < 100 k residents	1578 (64.5%)	1.06 [1.00, 1.12]	0.041	971 (77.7%)	1.03 [0.96, 1.11]	0.397
Town 100 k—< 1 million residents	1289 (61.0%)	1.00 [0.95, 1.06]	0.988	578 (75.1%)	1.00 [0.92, 1.08]	0.980
Major city ≥ 1 million residents	1194 (61.1%)	1		356 (75.6%)	1	

^1^*GISD* German Index of Socioeconomic Deprivation, ^2^*MSM* Men who have sex with men, ^3^*HET* Persons with heterosexual contact

^†^Strata specific number and proportion of non-recent infections at the time of diagnosis

^‡^Prevalence ratios with corresponding 95% confidence intervals of non-recent infections at the time of diagnosis were calculated for the exposure variable GISD using stratified multivariable Poisson regression with ZIP code cluster-robust error variance (standard errors were adjusted for 626 clusters in the MSM stratum and 562 clusters in the HET stratum). The models were adjusted for the variables of sex, approximated age at the time of infection, region of origin and city size

**Table 3:** Multivariable analysis of non-recent HIV infections including interaction term between GISD^1^ and city size (only MSM^2^)

	n = 6511					
	Countryside (< 100 k residents)			Town/Major city (≥ 100 k residents)		
	n (%)^†^	aPR [95% CI]^‡^	*p*-value	n (%)^†^	aPR [95% CI]^‡^	*p*-value
GISD^1^						
Low deprivation	234 (59.9%)	1		815 (62.2%)	1	
Medium deprivation	1090 (64.7%)	1.09 [1.01, 1.18]	0.033	1521 (60.4%)	0.97 [0.92, 1.03]	0.350
High deprivation	251 (68.8%)	1.16 [1.05, 1.28]	0.004	147 (61.8%)	1.00 [0.92, 1.09]	0.953

^1^*GISD* German Index of Socioeconomic Deprivation, ^2^*MSM* Men who have sex with men

^†^Strata specific number and proportion of non-recent infections at the time of diagnosis

^‡^Prevalence ratios with corresponding 95% confidence intervals of non-recent infections at the time of diagnosis were calculated for the exposure variable GISD using multivariable Poisson regression with ZIP code cluster-robust error variance (standard errors were adjusted for 626 clusters). The model was stratified for MSM and adjusted for the variables of approximated age at the time of infection, region of origin and city size. For simplicity purposes, only strata specific effect estimates of the GISD conditional on countryside vs. town/major city are depicted. The effect estimates of the remaining covariates are nearly identical as presented in Table 2 in the MSM stratum

In the stratified multivariable analyses of the CDC outcome measure, a total of 8198 MSM and 3113 heterosexual persons (2099 women and 1014 men) were included (see Additional file 2: Figure S2). Among MSM living in regions belonging to the highest socioeconomic deprivation quintile, we observed a weak increase in the prevalence of AIDS at the time of diagnosis (aPR: 1.21, 95% CI: 0.98–1.50) compared to MSM in regions of lower deprivation (Table 4). In the heterosexual transmission group, again no differences between persons of high, medium or low socioeconomic deprivation background were observed (Table 4). In this analysis, an interaction term between the GISD and city size was also included in the regression model of the MSM stratum, which revealed an interaction of the same type between these variables. While MSM living in highly deprived countryside areas had a significant increase in the prevalence of AIDS at the time of their diagnosis (aPR: 1.41, 95% CI: 1.08–1.85) compared to MSM who live in less deprived countryside areas (Table 5), no meaningful differences were observed among MSM from towns or major cities with different deprivation levels (Table 5).

**Table 4:** Multivariable analysis of infections at the stage of AIDS^1^ stratified by transmission mode

	n = 8198			n = 3113		
	MSM^3^			HET^4^		
	n (%)^†^	aPR [95% CI]^‡^	*p*-value	n (%)^†^	aPR [95% CI]^‡^	*p*-value
GISD^2^						
Low deprivation	253 (11.4%)	1		149 (16.5%)	1	
Medium deprivation	594 (11.5%)	0.98 [0.85, 1.13]	0.750	299 (16.2%)	0.99 [0.82, 1.19]	0.923
High deprivation	126 (15.6%)	1.21 [0.98, 1.50]	0.076	61 (16.8%)	1.05 [0.79, 1.40]	0.735
Sex						
Male	973 (11.9%)	1		195 (19.2%)	1	
Female		(omitted)		314 (15.0%)	0.72 [0.60, 0.86]	< 0.001
Approx. age (time of infection)						
15 to 19	4 (3.5%)	0.26 [0.10, 0.89]	0.007	4 (5.8%)	0.40 [0.16, 1.02]	0.056
20 to 29	350 (12.1%)	0.94 [0.82, 1.07]	0.369	201 (19.0%)	1.31 [1.07, 1.60]	0.008
30 to 39	320 (12.8%)	1		155 (15.0%)	1	
40 to 49	218 (11.6%)	0.89 [0.76, 1.05]	0.185	90 (16.3%)	1.00 [0.78, 1.27]	0.975
50 to 59	66 (10.5%)	0.80 [0.62, 1.03]	0.082	46 (15.9%)	0.95 [0.68, 1.33]	0.761
60 to 69	15 (9.8%)	0.74 [0.45, 1.22]	0.243	12 (13.4%)	0.78 [0.45, 1.35]	0.371
> 69	0 (0.0%)	(omitted)		1 (4.6%)	0.25 [0.04, 1.75]	0.165
Region of origin						
Western and Central Europe	896 (12.0%)	1		215 (17.5%)	1	
Asia and the Pacific	30 (14.9%)	1.26 [0.90, 1.76]	0.173	35 (26.3%)	1.57 [1.16, 2.12]	0.003
Caribbean	1 (3.7%)	0.31 [0.05, 2.15]	0.238	3 (15.8%)	0.90 [0.33, 2.49]	0.846
Eastern Europe and Central Asia	13 (12.6%)	1.05 [0.64, 1.71]	0.849	26 (15.3%)	0.90 [0.62, 1.31]	0.596
Latin America	15 (7.0%)	0.62 [0.38, 1.02]	0.059	5 (19.2%)	1.03 [0.47, 2.29]	0.935
Middle East and North Africa	5 (5.3%)	0.44 [0.19, 1.02]	0.057	11 (14.1%)	0.71 [0.38, 1.32]	0.278
North America	4 (9.8%)	0.88 [0.36, 2.17]	0.789	1 (25.0%)	1.45 [0.23, 9.01]	0.692
Sub-Saharan Africa	9 (12.9%)	1.08 [0.58, 2.01]	0.798	213 (14.7%)	0.79 [0.66, 0.95]	0.014
City size						
Countryside < 100 k residents	426 (14.1%)	1.37 [1.16, 1.64]	< 0.001	253 (16.1%)	0.90 [0.73, 1.11]	0.333
Town 100 k—< 1 million residents	287 (11.4%)	1.13 [0.94, 1.35]	0.192	150 (16.2%)	0.94 [0.75, 1.17]	0.568
Major city > = 1 million residents	260 (9.9%)	1		106 (17.4%)	1	

^1^*AIDS* Evidence of AIDS-defining illness, ^2^*GISD* German Index of Socioeconomic Deprivation, ^3^*MSM* Men who have sex with men, ^4^*HET* Persons with heterosexual contact

^†^Strata specific number and proportion of infections at the stage of AIDS at the time of diagnosis

^‡^Prevalence ratios with corresponding 95% confidence intervals of infections at the stage of AIDS at the time of diagnosis were calculated for the exposure variable GISD using stratified multivariable Poisson regression with ZIP code cluster-robust error variance (standard errors were adjusted for 644 clusters in the MSM stratum and 592 clusters in the HET stratum). The models were adjusted for the variables of sex, approximated age at the time of infection, region of origin and city size

**Table 5:** Multivariable analysis of infections at the stage of AIDS^1^ including interaction term between GISD^2^ and city size (only MSM^3^)

n = 8198						
	Countryside (< 100 k residents)			Town/Major city (> = 100 k residents)		
	n (%)^†^	aPR [95% CI]^‡^	*p*-value	n (%)^†^	aPR [95% CI]^‡^	*p*-value
GISD^1^						
Low deprivation	66 (12.3%)	1		187 (11.1%)	1	
Medium deprivation	268 (13.7%)	1.12 [0.88, 1.42]	0.373	326 (10.2%)	0.92 [0.77, 1.10]	0.348
High deprivation	92 (17.2%)	1.41 [1.08, 1.85]	0.013	34 (12.5%)	1.12 [0.75, 1.68]	0.587

^1^*AIDS* Evidence of AIDS-defining illness, ^2^*GISD* German Index of Socioeconomic Deprivation, ^3^*MSM* Men who have sex with men

^†^Strata specific number and proportion of infections at the stage of AIDS at the time of diagnosis

^‡^Prevalence ratios with corresponding 95% confidence intervals of infections at the stage of AIDS at the time of diagnosis were calculated for the exposure variable GISD using multivariable Poisson regression with ZIP code cluster-robust error variance (standard errors were adjusted for 644 clusters). The model was stratified for MSM and adjusted for the variables of approximated age at the time of infection, region of origin and city size. For simplicity purposes, only strata specific effect estimates of the GISD conditional on countryside vs. town/major city are depicted. The effect estimates of the remaining covariates are nearly identical as presented in Table 4 in the MSM stratum

Our sensitivity analyses to address a potential high proportion of migrants among the heterosexual transmission group showed that for both outcome variables, more than half of the persons with heterosexual contact originated from regions with differing HIV prevalences and dynamics, mainly from Sub-Saharan Africa (see Additional file 4: Tables S3 and S4). The post-hoc stratified multivariable analyses restricting the population to people of Western and Central European origin however yielded similar results to the main analyses (see Additional file 4: Tables S5–S8).

## Discussion

This study investigated the impact of regional socioeconomic deprivation on the timing of HIV diagnoses in Germany. The proportion of non-recent infections as well as AIDS at the time of diagnosis was somewhat higher in regions of high socioeconomic deprivation compared to regions with low and medium deprivation. The results of the multivariable analyses showed that MSM living in highly deprived regions in the countryside were more likely to have a non-recent infection and also more likely to have already developed an AIDS-defining condition at the time of their diagnosis compared to MSM in less deprived countryside regions. This effect of deprivation was not observed among MSM who live in towns or major cities. Among persons who acquired HIV via heterosexual contact, no effect of regional socioeconomic deprivation on the timing of their diagnosis was observed. Since the proportion of non-recent and AIDS diagnoses was substantially higher in the heterosexual transmission group compared to MSM, the results suggest that persons with heterosexual contact are generally at a higher risk of presenting late irrespective of their socioeconomic background.

Previous studies in Germany have mainly focused on the distribution of late HIV presentation by available sociodemographic data of the national case surveillance [31]. The findings of our study are consistent with these as the highest proportions of non-recent and AIDS diagnoses were also observed among persons with heterosexual contact, older age at the time of diagnosis and migrants from high-prevalence countries [12, 13, 31]. These patterns have remained stable over time and were also observed in other Western European and North American countries [11, 32–34]. The relationship between limited socioeconomic resources and HIV has found attention especially in research conducted in the US where several studies found that the prevalence of advanced HIV infections was higher in impoverished areas and that structural inequalities drove later diagnoses [17, 35–37].

The results of our study confirm the findings of previous research in other Western contexts. The observation of later diagnoses being more prevalent in areas of high socioeconomic deprivation has been attributed to the fact that people with fewer socioeconomic resources generally have lower health literacy, are less likely to practice healthy behaviours and have worse access to treatment [36, 38, 39]. The findings of our study also build on existing evidence with regard to the other population groups who are more likely to be diagnosed at advanced stages of their HIV infection. It is assumed that persons with heterosexual contact often do not test for HIV due to a lower self-perception of risk as well as limited knowledge and stigma-related misconceptions about HIV [40]. At the same time, this low awareness of HIV in the heterosexual transmission group is also existent among health care professionals, who as a result often do not consider an HIV infection even if indicator illnesses are present [41]. In addition to that, we observed that a high proportion of the persons who acquired HIV via heterosexual contact originated from regions outside of Western and Central Europe, mainly from Sub-Saharan Africa. This population group has increasingly migrated to Germany after 2013 and often faces greater barriers to HIV testing due to fears of asylum rejection and stigmatisation [12, 42]. As also confirmed by our sensitivity analyses, this higher risk of being diagnosed at later stages among persons with heterosexual contact can be observed in both migrant and non-migrant populations.

Regarding the impact of socioeconomic deprivation, our study’s findings provide new insights into the differences between the main transmission groups as well as the role of city size. After having controlled for confounding variables, we were able to identify an effect of socioeconomic deprivation in MSM from countryside areas but not in MSM from towns or bigger cities, and on the whole not in persons with heterosexual contact. A possible hypothesis might be that socioeconomic factors that are usually associated with health outcomes do not play a role among persons with heterosexual contact because risk and stigma-related misconceptions outweigh the expected impact of socioeconomic inequalities. Seeing that MSM have been and still are the population group most affected by HIV in the Western context, HIV education traditionally had a stronger focus on MSM [13]. This might explain why MSM generally have a lower risk of presenting late but are affected by a lack of socioeconomic resources when it comes to HIV health outcomes. As the separate assessment of MSM who live in countryside versus urban areas however revealed that only MSM from the countryside were affected by socioeconomic deprivation, it can be assumed that urban areas offer better support structures for this group. The fact that HIV checkpoints, specialised medical practices and MSM communities are more often located in towns and bigger cities [19, 43, 44] might help to offset the impact of socioeconomic deprivation for MSM in urban areas when it comes to the detection of HIV.

These findings should be taken into consideration when developing strategies aimed at reducing the number of HIV infections diagnosed at later stages in Germany. Socioeconomic status is a complex social construct which cannot be directly intervened on. It therefore has to be regarded and interpreted rather as a proxy for a wide range of associated factors, which in the context of our analyses includes factors such as health literacy and awareness, healthy behaviours, lifestyle and access to medical services [45]. The results suggest that public health response should target persons with heterosexual contact irrespective of their socioeconomic background. As missed opportunities for early diagnoses occur particularly in this population group [46], increased efforts in sensitising general practitioners for HIV among heterosexual persons could prove effective in capturing more undiagnosed infections. Especially women’s health care professionals could play a key role, seeing that women accounted for the majority of the newly diagnosed HIV infections in the heterosexual transmission group. In addition to that, increased opportunistic screening and efforts in reducing stigma-related beliefs could facilitate early HIV detection among migrants from high-prevalence countries. When it comes to MSM, our results suggest that structural inequalities should be considered with a focus on rural regions of higher socioeconomic deprivation. Here, public health efforts could address the expansion of specialised medical practices for HIV as well as HIV checkpoints in these regions in order to increase awareness and offer more low-barrier testing opportunities for MSM. Since our study offers new insights into the relationship of regional socioeconomic deprivation and late HIV presentation in the context of Germany, we encourage further research that builds on these findings. In-depth investigations into which HIV health behaviours as well as structural factors related to the healthcare system are most affected by socioeconomic deprivation could help to further improve the public health response.

## Strengths and limitations

A strength of this study is the utilisation of the GISD in the analysis of the impact of regional socioeconomic deprivation. Since the score was calculated for each region nationwide, it was possible to analyse the majority of newly diagnosed HIV infections reported between 2011 and 2018 with available BED-CEIA results and information on CDC classification. In addition to that, the GISD has shown to be a reliable tool for the analysis of regional socioeconomic inequalities and health as statistical links were also found in prior research concerning life expectancy, major causes of death and various behavioural health risks in Germany [18, 47].

This study is also subject to limitations. As the BED-CEIA produces false-recent results with differing rates across HIV subtypes [24] and false-recent results were corrected only on the basis of AIDS diagnoses, the prevalence of non-recent infections was probably underestimated in our analyses. Due to the fact that the FRR is significantly higher among non-B subtypes which are more prevalent outside of Western Europe [24, 48], an underestimation of non-recent diagnoses presumably affected the migrant sub-populations in our analyses more. However, as the sensitivity analyses which were restricted to people from Western and Central Europe showed similar results, we believe that our analyses and main findings were not substantially impacted by this factor. Another limitation is the high proportion of missing values in both outcome variables, resulting from the fact that the CDC classification was not always indicated and dried blood spots which can be tested for recency were only received from laboratories that have agreed to participate. The conducted sensitivity analyses however showed that included and excluded persons were generally comparable with regard to baseline characteristics, allowing for the assumption that our analyses were not considerably affected by the missing data. A further limitation to be mentioned is our approach of estimating the variable age at the time of infection, which is crude and can therefore only represent a rough approximation. Lastly, the GISD illustrates regional and not individual socioeconomic deprivation. It is therefore beyond the scope of this study to make claims about the impact of a person’s individual socioeconomic status on late HIV presentation. Nevertheless, as risk factors regarding health outcomes are generally more prevalent in areas characterised by high levels of socioeconomic deprivation, regional socioeconomic factors have proven to serve as valid proxy variables for aggregated individual socioeconomic deprivation [18].

## Conclusions

Our study has found that HIV infections which are diagnosed at non-recent and AIDS stages are somewhat more prevalent in areas characterised by high levels of socioeconomic deprivation. After having controlled for confounding variables, an effect of socioeconomic deprivation on the timing of HIV diagnosis was however only found among MSM from countryside areas. In order to tackle the burden of late HIV presentation in Germany, the findings suggest that efforts in promoting HIV awareness and regular testing behaviours have to be increased for heterosexual persons irrespective of their socioeconomic background. For MSM, we recommend that a focus is put on those living in rural regions of higher socioeconomic deprivation.

## Supplementary Information

**Additional file 1: Figure S1.** Directed acyclic graph (DAG).

**Additional file 2: Figure S2.** Flowchart.

**Additional file 3: Tables S1 and S2.** Sensitivity analyses.

**Additional file 4: Tables S3–S8.** Sensitivity analyses.

## Data Availability

The HIV data analysed during the current study are not publicly available. They were generated from the national statutory notification of newly diagnosed HIV cases and blood samples which are directly sent and processed at the Robert Koch Institute. The dataset is available from the corresponding author on reasonable request. The German Index of Socioeconomic Deprivation is publicly available and can be accessed via the GESIS repository, https://doi.org/10.7802/1460.
