# Supplementary material for: The impact of regional socioeconomic deprivation on the timing of HIV diagnosis: a cross-sectional study in Germany
Source: BMC Infect Dis. 2022 Mar 17;22:258. doi: 10.1186/s12879-022-07168-x (PMC8928640; doi:10.1186/s12879-022-07168-x)
Supplement: Supplementary file 1 — Additional file 1: Figure S1. Directed acyclic graph (DAG). [file 12879_2022_7168_MOESM1_ESM.docx]

**Additional file 1**

Figure S1: Directed acyclic graph (DAG)

Word format. The DAG models the relationships of relevant variables and was used to identify the minimally sufficient adjustment set of confounders.


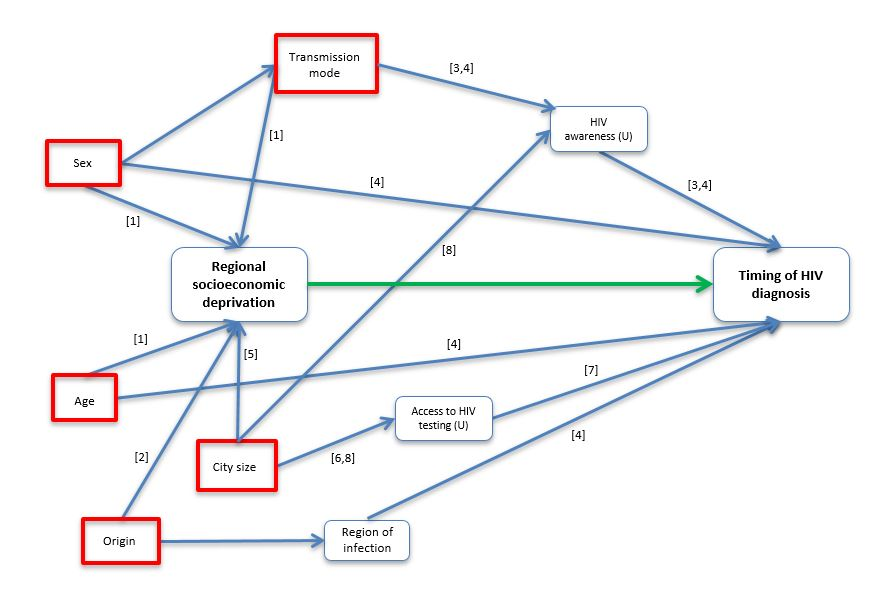


**Figure S1: Directed acyclic graph (DAG)**

This DAG displays the assumptions about the relationships between the variables that are involved in the relationship of interest, which is the effect of regional socioeconomic deprivation (exposure variable) on the timing of HIV diagnosis (outcome variable). Unmeasured variables are labelled with (U). The assumptions were based on prior research (numbers on the paths depict references) or general knowledge. As we were only interested in the causal path from regional socioeconomic deprivation to timing of HIV diagnosis, all “open back-door” paths needed to be “closed”. Open back-door paths equal confounding and have to be accounted for in the analysis. The minimally sufficient adjustment set of variables was identified, which when adjusted for blocks all open back-door paths and includes no more variables than necessary.

The identified confounding variables were transmission mode, sex, age, region of origin and city size and were controlled for in the analyses. With regard to the variable of age, we decided to use the computed approximated age at the time of infection instead of the age at the time of diagnosis. In previous research it is often reported that older age is linked to late HIV presentation [9,10], using age at the time of diagnosis. This does not take into consideration the fact that persons who were diagnosed after they had developed an AIDS-defining condition have aged by a median period of ten years compared to those who tested for HIV in the months following their contraction [11]. Hence, utilising the approximated age at infection allowed to account for the fact that the longer an infection remains undiagnosed, the more time passes and the older the individual becomes.

**References**

1. Lampert T, Kroll LE, Müters S, Stolzenberg H. Measurement of the socioeconomic status within the German Health Update 2009 (GEDA). Bundesgesundheitsbl. 2013;56:131-43.
2. De Groot O, Sagner L. Migranten in Deutschland: Soziale Unterschiede hemmen Integration. Deutsches Institut für Wirtschaftsforschung; 2010.
3. Matl M. HIV: Späte Diagnose bei Heterosexuellen. Österreichischer Rundfunk. 2019. https://noe.orf.at/stories/3023018/. Accessed 27 Mar 2020.
4. Hofmann A, Hauser A, Zimmermann R, Santos-Hövener C, Bätzing-Feigenbaum J, Wildner S, et al. Surveillance of recent HIV infections among newly diagnosed HIV cases in Germany between 2008 and 2014. BMC Infect Dis. 2017;17(484).
5. Kroll LE, Schumann M, Hoebel J, Lampert T. Regionale Unterschiede in der Gesundheit - Entwicklung eines sozioökonomischen Deprivationsindex für Deutschland. J Health Monit. 2017;2(2):103-20.
6. Etgeton S. Ärztedichte: Neue Bedarfsplanung geht am Bedarf vorbei. Spotlight Gesundheit. 2015.
7. Wurm M, Neumann A, Wasem J, Biermann-Stallwitz J. Zugangsschwellen zu HIV-Test-Angeboten – Eine systematische Literaturanalyse. Gesundheitswesen. 2019;81(3):e43-e57.
8. Mau M. Das Landei: HIV-Versorgung außerhalb der Großstädte. Esanum. 2018. https://www.esanum.de/fachbereichsseite-dermatologie/feeds/dermatologie/conferences/AIDS-Hepatitis-18/feeds/today/posts/das-landei-hiv-versorgung-auerhalb-der-grostadte. Accessed 27 Mar 2020.
9. Hu X, Liang B, Zhou C, Jiang J, Huang J, Ning C, et al. HIV late presentation and advanced HIV disease among patients with newly diagnosed HIV/AIDS in Southwestern China: a large-scale cross-sectional study. AIDS Res Ther. 2019;16(1):6.
10. Mugavero MJ, Castellano C, Edelman D, Hicks C. Late diagnosis of HIV infection: the role of age and sex. Am J Med. 2007;120(4):370-3.
11. Collaborative Group on AIDS Incubation and HIV Survival including the CASCADE EU Concerted Action. Time from HIV-1 seroconversion to AIDS and death before widespread use of highly-active antiretroviral therapy: a collaborative re-analysis. Lancet. 2000;355(9210):1131-7.
