# Supplementary material for: The impact of regional socioeconomic deprivation on the timing of HIV diagnosis: a cross-sectional study in Germany
Source: BMC Infect Dis. 2022 Mar 17;22:258. doi: 10.1186/s12879-022-07168-x (PMC8928640; doi:10.1186/s12879-022-07168-x)
Supplement: Supplementary file 2 — Additional file 2: Figure S2. Flowchart. [file 12879_2022_7168_MOESM2_ESM.docx]

**Additional file 2**

Figure S2: Flowchart

Word format. The flowchart depicts the inclusion/exclusion steps of newly diagnosed HIV infections in our analyses that were reported in Germany between 2011 and 2018.


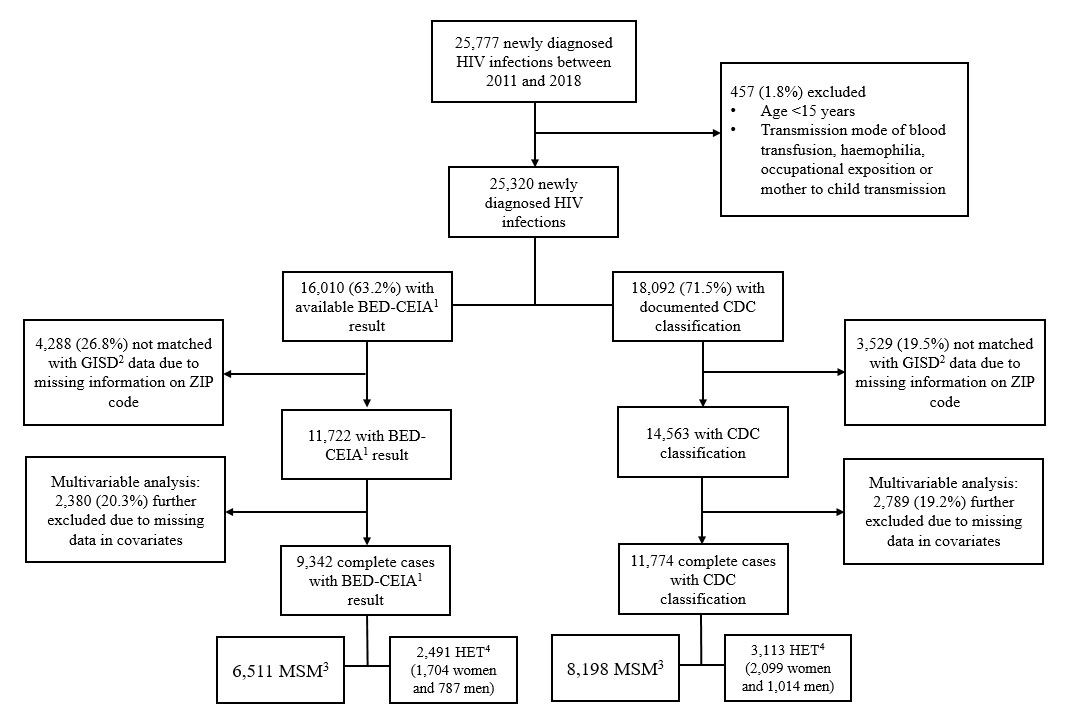
**Figure S2: Flowchart of newly diagnosed HIV infections in Germany between 2011 and 2018 included in the analyses**

^1^*BED-CEIA* BED-Capture-ELISA recency test, ^2^*GISD* German Index of Socioeconomic Deprivation, ^3^*MSM* Men who have sex with men, ^4^*HET* Persons

with heterosexual contact
