# Supplementary material for: The impact of regional socioeconomic deprivation on the timing of HIV diagnosis: a cross-sectional study in Germany
Source: BMC Infect Dis. 2022 Mar 17;22:258. doi: 10.1186/s12879-022-07168-x (PMC8928640; doi:10.1186/s12879-022-07168-x)
Supplement: Supplementary file 3 — Additional file 3: Tables S1 and S2. Sensitivity analyses. [file 12879_2022_7168_MOESM3_ESM.docx]

**Additional file 3**

Tables S1 and S2: Sensitivity analyses

Word format. The charts depict the distribution of baseline characteristics of cases with available vs. missing data in the outcome measures.

| **Table S1 Comparison of baseline characteristics between persons with available and missing data in BED-CEIA^1^ outcome**  **N=25,320** | | |
| --- | --- | --- |
|  | available | missing |
| Total | 16,010 (100.0%) | 9,310 (100.0%) |
| GISD^2^ |  |  |
| Low deprivation | 3,116 (19.5%) | 2,115 (22.7%) |
| Medium deprivation | 7,396 (46.2%) | 4,671 (50.2%) |
| High deprivation | 1,210 (7.6%) | 1,088 (11.7%) |
| Missing | 4,288 (26.8%) | 1,436 (15.4%) |
| Transmission mode |  |  |
| MSM^3^ | 8,879 (55.5%) | 4,806 (51.6%) |
| HET^4^ | 3,844 (24.0%) | 2,034 (21.9%) |
| PWID^5^ | 548 (3.4%) | 384 (4.1%) |
| Missing | 2,739 (17.1%) | 2,086 (22.4%) |
| Sex |  |  |
| Male | 12,923 (80.7%) | 7,598 (81.6%) |
| Female | 3,065 (19.1%) | 1,693 (18.2%) |
| Missing | 21 (0.1%) | 19 (0.2%) |
| Age (time of diagnosis) |  |  |
| 15 to 19 | 303 (1.9%) | 135 (1.5%) |
| 20 to 29 | 4,303 (26.8%) | 2,237 (24.0%) |
| 30 to 39 | 5,080 (31.7%) | 2,856 (30.7%) |
| 40 to 49 | 3,647 (22.8%) | 2,280 (24.5%) |
| 50 to 59 | 1,869 (11.7%) | 1,223 (13.1%) |
| 60 to 69 | 562 (3.5%) | 401 (4.3%) |
| >69 | 179 (0.1%) | 126 (1.4%) |
| Missing | 67 (0.4%) | 52 (0.6%) |
| Approx. age (time of infection) |  |  |
| 15 to 19 | 303 (1.9%) | 135 (1.5%) |
| 20 to 29 | 5,853 (36.6%) | 2,889 (31.0%) |
| 30 to 39 | 4,865 (30.4%) | 2,865 (30.8%) |
| 40 to 49 | 3,220 (20.1%) | 2,104 (22.6%) |
| 50 to 59 | 1,253 (7.8%) | 924 (9.9%) |
| 60 to 69 | 363 (2.3%) | 273 (2.9%) |
| >69 | 86 (0.5%) | 68 (0.7%) |
| Missing | 67 (0.4%) | 52 (0.6%) |
| Region of origin |  |  |
| Western and Central Europe | 10,922 (68.2%) | 6,570 (70.6%) |
| Asia and the Pacific | 466 (2.9%) | 222 (2.4%) |
| Caribbean | 71 (0.4%) | 28 (0.3%) |
| Eastern Europe and Central Asia | 668 (4.2%) | 337 (3.6%) |
| Latin America | 337 (2.1%) | 160 (1.7%) |
| Middle East and North Africa | 348 (2.2%) | 179 (1.9%) |
| North America | 68 (0.4%) | 39 (0.4%) |
| Sub-Saharan Africa | 2,205 (13.8%) | 1,045 (11.2%) |
| Missing | 925 (5.8%) | 730 (7.8%) |
| City size |  |  |
| Countryside <100k res. | 6,319 (39.5%) | 3,598 (38.6%) |
| Town 100k - <1 million res. | 5,161 (32.2%) | 2,995 (32.2%) |
| Major city >= 1 million res. | 4,481 (28.0%) | 2,656 (28.5%) |
| Missing | 49 (0.3%) | 61 (0.7%) |
| Numbers may not add up to 100% because of rounding  ^1^*BED-CEIA* BED-Capture-ELISA recency test, ^2^*GISD* German Index of Socioeconomic Deprivation, | | |
| ^3^*MSM* Men who have sex with men, ^4^*HET* Persons with heterosexual contact, ^5^*PWID* Persons who inject drugs | | |

| **Table S2 Comparison of baseline characteristics between persons with available and missing data in CDC classification outcome**  **N=25,320** | | |
| --- | --- | --- |
|  | available | missing |
| Total | 18,092 (100.0%) | 7,228 (100.0%) |
| GISD^1^ |  |  |
| Low deprivation | 3,940 (21.8%) | 1,291 (17.9%) |
| Medium deprivation | 8,969 (49.6%) | 3,098 (42.9%) |
| High deprivation | 1,654 (9.1%) | 644 (8.9%) |
| Missing | 3,729 (20.6%) | 2,195 (30.4%) |
| Transmission mode |  |  |
| MSM^2^ | 10,162 (56.2%) | 3,523 (48.7%) |
| HET^3^ | 4,252 (23.5%) | 1,626 (22.5%) |
| PWID^4^ | 678 (3.7%) | 254 (3.5%) |
| Missing | 3,000 (16.6%) | 1,825 (25.3%) |
| Sex |  |  |
| Male | 14,729 (81.4%) | 5,792 (80.1%) |
| Female | 3,353 (18.5%) | 1,405 (19.4%) |
| Missing | 10 (<0.1%) | 31 (0.4%) |
| Age (time of diagnosis) |  |  |
| 15 to 19 | 287 (1.6%) | 151 (2.1%) |
| 20 to 29 | 4,482 (24.8%) | 2,058 (28.5%) |
| 30 to 39 | 5,638 (31.2%) | 2,298 (31.8%) |
| 40 to 49 | 4,364 (24.1%) | 1,563 (21.6%) |
| 50 to 59 | 2,309 (12.8%) | 783 (10.8%) |
| 60 to 69 | 746 (4.1%) | 217 (3.0%) |
| >69 | 218 (1.2%) | 87 (1.2%) |
| Missing | 48 (0.3%) | 71 (1.0%) |
| Approx. age (time of infection) |  |  |
| 15 to 19 | 287 (1.6%) | 151 (2.1%) |
| 20 to 29 | 6,121 (33.8%) | 2,612 (36.3%) |
| 30 to 39 | 5,626 (31.1%) | 2,104 (29.1%) |
| 40 to 49 | 3,896 (21.5%) | 1,428 (19.8%) |
| 50 to 59 | 1,566 (8.6%) | 611 (8.5%) |
| 60 to 69 | 460 (2.5%) | 176 (2.4%) |
| >69 | 88 (0.5%) | 66 (0.9%) |
| Missing | 48 (0.3%) | 71 (1.0%) |
| Region of origin |  |  |
| Western and Central Europe | 13,094 (72.4%) | 4,398 (60.9%) |
| Asia and the Pacific | 501 (2.8%) | 187 (2.6%) |
| Caribbean | 71 (3.9%) | 28 (0.3%) |
| Eastern Europe and Central Asia | 725 (4.0%) | 280 (3.9%) |
| Latin America | 365 (2.0%) | 132 (1.8%) |
| Middle East and North Africa | 369 (2.0%) | 158 (2.2%) |
| North America | 67 (0.4%) | 40 (0.6%) |
| Sub-Saharan Africa | 2,223 (12.3%) | 1,027 (14.2%) |
| Missing | 677 (3.7%) | 978 (13.5%) |
| City size |  |  |
| Countryside <100k res. | 7,295 (40.3%) | 2,622 (36.3%) |
| Town 100k - <1 million res. | 5,716 (31.6%) | 2,440 (33.8%) |
| Major city >= 1 million res. | 5,016 (27.7%) | 2,121 (29.3%) |
| Missing | 65 (0.4%) | 45 (0.6%) |
| Numbers may not add up to 100% because of rounding  ^1^*GISD* German Index of Socioeconomic Deprivation, ^2^*MSM* Men who have sex with men, ^3^*HET* Persons  with heterosexual contact, ^4^*PWID* Persons who inject drugs | | |
