# Supplementary material for: The impact of regional socioeconomic deprivation on the timing of HIV diagnosis: a cross-sectional study in Germany
Source: BMC Infect Dis. 2022 Mar 17;22:258. doi: 10.1186/s12879-022-07168-x (PMC8928640; doi:10.1186/s12879-022-07168-x)
Supplement: Supplementary file 4 — Additional file 4: Tables S3–S8. Sensitivity analyses. [file 12879_2022_7168_MOESM4_ESM.docx]

**Additional file 4**

Tables S3-S8: Sensitivity analyses

Word format. Tables S3 and S4 depict the descriptive analyses conducted to address the issue of a potential high proportion of migrants among the heterosexual transmission group. Tables S5-S8 depict the multivariable analyses restricted to persons from Western and Central Europe only to examine whether similar results are yielded if migrants from regions with differing HIV prevalences and dynamics are excluded.

| **Table S3 Distribution of region of origin among HET^1^ with available BED-CEIA^2^ result** | |
| --- | --- |
|  | **n (%)** |
| Total | 2,491 (100.0%) |
| Region of origin |  |
| Western and Central Europe | 891 (35.8%) |
| Asia and the Pacific | 109 (4.4%) |
| Caribbean | 16 (0.6%) |
| Eastern Europe and Central Asia | 141 (5.7%) |
| Latin America | 19 (0.8%) |
| Middle East and North Africa | 67 (2.7%) |
| North America | 2 (0.1%) |
| Sub-Saharan Africa | 1,246 (50.0%) |
| Numbers may not add up to 100% because of rounding  ^1^*HET* Persons with heterosexual contact, ^2^*BED-CEIA* BED-  Capture-ELISA recency test | |

| **Table S4 Distribution of region of origin among HET^1^ with documented CDC classification** | |
| --- | --- |
|  | **n (%)** |
| Total | 3,113 (100.0%) |
| Region of origin |  |
| Western and Central Europe | 1,229 (39.5%) |
| Asia and the Pacific | 133 (4.3%) |
| Caribbean | 19 (0.6%) |
| Eastern Europe and Central Asia | 170 (5.5%) |
| Latin America | 26 (0.8%) |
| Middle East and North Africa | 78 (2.5%) |
| North America | 4 (0.1%) |
| Sub-Saharan Africa | 1,454 (46.7%) |
| Numbers may not add up to 100% because of rounding  ^1^*HET* Persons with heterosexual contact | |

| **Table S5 Multivariable analysis of non-recent HIV infections stratified by transmission mode among persons from Western and Central Europe** | | | | | | | | | | | | | |  |
| --- | --- | --- | --- | --- | --- | --- | --- | --- | --- | --- | --- | --- | --- | --- |
| **n=5,926 n=891** | | | | | | | | | | | | | |  |
|  | |  | | **MSM^2^** | |  | |  | | **HET^3^** | |  | |  |
|  | | **n (%)**† | | **aPR [95% CI]‡** | | ***p*-value** | | **n (%)**† | | **aPR [95% CI]‡** | | ***p*-value** | |  |
| GISD^1^ | |  |  | | |  |  | | |  | |  | |  |
| Low deprivation | | 940 (61.3%) | 1 | | |  | 158 (71.2%) | | | 1 | |  | |  |
| Medium deprivation | | 2,368 (62.0%) | 1.01 [0.96, 1.06] | | | 0.736 | 404 (71.1%) | | | 1.00 [0.90, 1.10] | | 0.932 | |  |
| High deprivation | | 377 (65.6%) | 1.06 [0.99, 1.14] | | | 0.096 | 69 (68.3%) | | | 0.96 [0.81, 1.14] | | 0.662 | |  |
| Sex | |  |  | | |  |  | | |  | |  | |  |
| Male | | 3,685 (62.2%) | 1 | | |  | 205 (70.2%) | | | 1 | |  | |  |
| Female | |  | (omitted) | | |  | 426 (71.1%) | | | 1.02 [0.93, 1.12] | | 0.673 | |  |
| Approx. age (time of infection) | |  |  | | |  |  | | |  | |  | |  |
| 15 to 19 | | 39 (37.1%) | 0.59 [0.46, 0.75] | | | <0.001 | 9 (47.4%) | | | 0.67 [0.41, 1.09] | | 0.106 | |  |
| 20 to 29 | | 1,282 (61.1%) | 0.97 [0.92, 1.02] | | | 0.250 | 184 (72.2%) | | | 1.02 [0.91, 1.15] | | 0.737 | |  |
| 30 to 39 | | 1,116 (62.7%) | 1 | | |  | 173 (70.6%) | | | 1 | |  | |  |
| 40 to 49 | | 904 (66.2%) | 1.05 [1.00, 1.11] | | | 0.047 | 130 (69.9%) | | | 0.99 [0.87, 1.13] | | 0.911 | |  |
| 50 to 59 | | 256 (59.5%) | 0.94 [0.87, 1.02] | | | 0.165 | 95 (74.8%) | | | 1.06 [0.93, 1.21] | | 0.380 | |  |
| 60 to 69 | | 74 (63.8%) | 1.01 [0.87, 1.19] | | | 0.862 | 30 (68.2%) | | | 0.97 [0.79, 1.20] | | 0.788 | |  |
| >69 | | 14 (50.0%) | 0.80 [0.57, 1.11] | | | 0.145 | 10 (66.7%) | | | 0.95 [0.65, 1.39] | | 0.789 | |  |
| City size | |  |  | | |  |  | | |  | |  | |  |
| Countryside <100k res. | | 1,476 (64.2%) | 1.05 [0.99, 1.11] | | | 0.116 | 324 (71.8%) | | | 1.05 [0.91, 1.20] | | 0.527 | |  |
| Town 100k - <1 million res. | | 1,145 (60.7%) | 0.99 [0.93, 1.05] | | | 0.733 | 197 (70.1%) | | | 1.03 [0.89, 1.18] | | 0.702 | |  |
| Major city >= 1 million res. | | 1,064 (61.2%) | 1 | | |  | 110 (69.2%) | | | 1 | |  | |  |
| ^1^*GISD* German Index of Socioeconomic Deprivation, ^2^*MSM* Men who have sex with men, ^3^*HET* Persons with heterosexual contact  †Strata specific number and proportion of non-recent infections at the time of diagnosis.  ‡Prevalence ratios with corresponding 95% confidence intervals of non-recent infections at the time of diagnosis were calculated for the exposure variable GISD using stratified multivariable Poisson regression with ZIP code cluster-robust error variance (standard errors were adjusted for 621 clusters in the MSM stratum and 393 clusters in the HET stratum). The models were adjusted for the variables of sex, approximated age at the time of infection and city size. | | | | | | | | | | | | | |  |
| **Table S6 Multivariable analysis of non-recent HIV infections including interaction term between GISD^1^ and city size (only MSM^2^ from Western and Central Europe)**  **n=5,926** | | | | | | | | | | | | | | |
|  | **Countryside (<100k residents)** | | | | | | | | **Town/Major city (>= 100k residents)** | | | | | |
|  | **n (%)†** | | **aPR [95% CI]‡** | | ***p*-value** | | | | **n (%)†** | | **aPR [95% CI]‡** | | ***p*-value** | |
| GISD^1^ |  | |  | |  | | | |  | |  | |  | |
| Low deprivation | 218 (59.2%) | | 1 | |  | | | | 722 (62.0%) | | 1 | |  | |
| Medium deprivation | 1,017 (64.5%) | | 1.09 [1.01, 1.19] | | 0.033 | | | | 1,351 (60.3%) | | 0.98 [0.92, 1.04] | | 0.440 | |
| High deprivation | 241 (68.5%) | | 1.17 [1.05, 1.30] | | 0.004 | | | | 136 (61.0%) | | 1.00 [0.91, 1.09] | | 0.918 | |
|  |  | |  | |  | | | |  | |  | |  | |
|  |  | |  | |  | | | |  | |  | |  | |
| ^1^*GISD* German Index of Socioeconomic Deprivation, ^2^*MSM* Men who have sex with men  †Strata specific number and proportion of non-recent infections at the time of diagnosis. | | | | | | | | | | | | | | |
| ‡Prevalence ratios with corresponding 95% confidence intervals of non-recent infections at the time of diagnosis were calculated for the exposure variable GISD using multivariable Poisson regression with ZIP code cluster-robust error variance (standard errors were adjusted for 621 clusters). The model was stratified for MSM from Western and Central Europe and adjusted for the variables of approximated age at the time of infection and city size. For simplicity purposes, only strata specific effect estimates of the GISD conditional on countryside vs. town/major city are depicted. The effect estimates of the remaining covariates are nearly identical as presented in Table S5 in the MSM stratum. | | | | | | | | | | | | | | |

| **Table S7 Multivariable analysis of infections at the stage of AIDS**^1^ **stratified by transmission mode among persons from Western and Central Europe** | | | | | | | | | | | | | |  |
| --- | --- | --- | --- | --- | --- | --- | --- | --- | --- | --- | --- | --- | --- | --- |
| **n=7,448 n=1,229** | | | | | | | | | | | | | |  |
|  | |  | | | **MSM^3^** |  | |  | | **HET^4^** | |  | |  |
|  | | **n (%)†** | | | **aPR [95% CI]‡** | ***p*-value** | | **n (%)†** | | **aPR [95% CI]‡** | | ***p*-value** | |  |
| GISD^2^ | |  |  | | |  |  | | |  | |  | |  |
| Low deprivation | | 228 (11.5%) | 1 | | |  | 55 (17.2%) | | | 1 | |  | |  |
| Medium deprivation | | 545 (11.6%) | 0.99 [0.85, 1.14] | | | 0.838 | 127 (17.1%) | | | 1.00 [0.76, 1.32] | | 0.998 | |  |
| High deprivation | | 123 (15.9%) | 1.24 [1.00, 1.54] | | | 0.050 | 33 (20.0%) | | | 1.20 [0.80, 1.79] | | 0.374 | |  |
| Sex | |  |  | | |  |  | | |  | |  | |  |
| Male | | 896 (12.0%) | 1 | | |  | 76 (18.3%) | | | 1 | |  | |  |
| Female | |  | (omitted) | | |  | 139 (17.1%) | | | 0.96 [0.73, 1.26] | | 0.762 | |  |
| Approx. age (time of infection) | |  |  | | |  |  | | |  | |  | |  |
| 15 to 19 | | 4 (3.8%) | 0.27 [0.10, 0.72] | | | 0.008 | 0 (0.0%) | | | (omitted) | |  | |  |
| 20 to 29 | | 305 (12.1%) | 0.91 [0.79, 1.04] | | | 0.174 | 52 (15.0%) | | | 0.83 [0.60, 1.15] | | 0.262 | |  |
| 30 to 39 | | 298 (13.2%) | 1 | | |  | 59 (18.1%) | | | 1 | |  | |  |
| 40 to 49 | | 212 (11.8%) | 0.89 [0.75, 1.06] | | | 0.181 | 61 (22.8%) | | | 1.24 [0.91, 1.69] | | 0.171 | |  |
| 50 to 59 | | 63 (10.6%) | 0.79 [0.60, 1.02] | | | 0.075 | 34 (18.5%) | | | 1.02 [0.67, 1.54] | | 0.936 | |  |
| 60 to 69 | | 14 (9.3%) | 0.69 [0.41, 1.17] | | | 0.170 | 9 (14.5%) | | | 0.78 [0.39, 1.54] | | 0.470 | |  |
| >69 | | 0 (0.0%) | (omitted) | | |  | 0 (0.0%) | | | (omitted) | |  | |  |
| City size | |  |  | | |  |  | | |  | |  | |  |
| Countryside <100k res. | | 401 (14.1%) | 1.37 [1.15, 1.64] | | | 0.001 | 111 (17.7%) | | | 1.04 [0.76, 1.41] | | 0.821 | |  |
| Town 100k - <1 million res. | | 261 (11.5%) | 1.12 [0.93, 1.34] | | | 0.222 | 64 (17.6%) | | | 1.05 [0.74, 1.49] | | 0.773 | |  |
| Major city >= 1 million res. | | 234 (10.0%) | 1 | | |  | 40 (16.9%) | | | 1 | |  | |  |
| ^1^*AIDS* Evidence of AIDS-defining illness, ^2^*GISD* German Index of Socioeconomic Deprivation, ^3^*MSM* Men who have sex with men, ^4^*HET* Persons with heterosexual contact  †Strata specific number and proportion of infections at the stage of AIDS at the time of diagnosis.  ‡Prevalence ratios with corresponding 95% confidence intervals of infections at the stage of AIDS at the time of diagnosis were calculated for the exposure variable GISD using stratified multivariable Poisson regression with ZIP code cluster-robust error variance (standard errors were adjusted for 643 clusters in the MSM stratum and 477 clusters in the HET stratum). The models were adjusted for the variables of sex, approximated age at the time of infection and city size. | | | | | | | | | | | | | |  |
| **Table S8 Multivariable analysis of infections at the stage of AIDS^1^ including interaction term between GISD^2^ and city size (only MSM^3^ from Western and Central Europe)**    **n=7,448** | | | | | | | | | | | | | | |
|  | **Countryside (<100k residents)** | | | | | | | | **Town/Major city (>= 100k residents)** | | | | | |
|  | **n (%)†** | | | **aPR [95% CI]‡** | | ***p*-value** | | | **n (%)†** | | **aPR [95% CI]‡** | | ***p*-value** | |
| GISD^1^ |  | | |  | |  | | |  | |  | |  | |
| Low deprivation | 60 (12.2%) | | | 1 | |  | | | 168 (11.2%) | | 1 | |  | |
| Medium deprivation | 251 (13.7%) | | | 1.13 [0.88, 1.46] | | 0.334 | | | 294 (10.3%) | | 0.92 [0.77, 1.10] | | 0.376 | |
| High deprivation | 90 (17.5%) | | | 1.46 [1.10, 1.94] | | 0.009 | | | 33 (12.6%) | | 1.13 [0.74, 1.72] | | 0.567 | |
|  |  | | |  | |  | | |  | |  | |  | |
|  |  | | |  | |  | | |  | |  | |  | |
| ^1^*AIDS* Evidence of AIDS-defining illness, ^2^*GISD* German Index of Socioeconomic Deprivation, ^3^*MSM* Men who have sex with men    †Strata specific number and proportion of infections at the stage of AIDS at the time of diagnosis. | | | | | | | | | | | | | | |
| ‡Prevalence ratios with corresponding 95% confidence intervals of infections at the stage of AIDS at the time of diagnosis were calculated for the exposure variable GISD using multivariable Poisson regression with ZIP code cluster-robust error variance (standard errors were adjusted for 643 clusters). The model was stratified for MSM from Western and Central Europe and adjusted for the variables of approximated age at the time of infection and city size. For simplicity purposes, only strata specific effect estimates of the GISD conditional on countryside vs. town/major city are depicted. The effect estimates of the remaining covariates are nearly identical as presented in Table S7 in the MSM stratum. | | | | | | | | | | | | | | |
